# QGQS Granule in SHR Serum Metabonomics Study Based on Tools of UPLC-Q-TOF and Renin-Angiotensin-Aldosterone System Form Protein Profilin-1

**DOI:** 10.1155/2017/4854720

**Published:** 2017-03-06

**Authors:** Ke Li, Caicai Li, Jialong Wang, Hanming Cui, Yu Dong, Ruihua Liu, Yuanhui Hu

**Affiliations:** Guang'anmen Hospital, China Academy of Chinese Medical Sciences, Beijing 100053, China

## Abstract

QGQS granule is effective for the therapeutic of hypertension in clinic. The aim of this research is to observe the antihypertension effect of QGQS granule on SHR and explain the mechanism of its lowering blood pressure. 30 SHR were selected as model group, captopril group, and QGQS group, 10 WKYr were used as control group, and RBP were measured on tail artery consciously. And all the serum sample analysis was carried out on UPLC-TOF-MS system to determine endogenous metabolites and to find the metabonomics pathways. Meanwhile, ELISA kits for the determination pharmacological indexes of PRA, AngI, AngII, and ALD were used for pathway confirmatory; WB for determination of profilin-1 protein expression was conducted for Ang II pathway analysis as well. It is demonstrated that QGQS granule has an excellent therapeutic effect on antihypertension, which exerts effect mainly on metabonomics pathway by regulating glycerophospholipid, sphingolipid, and arachidonic acid metabolism, and it could inhibit the overexpression of the profilin-1 protein. We can come to a conclusion that RAAS should be responsible mainly for the metabonomics pathway of QGQS granule on antihypertension, and it plays a very important role in protein of profilin-1 inhibition.

## 1. Introduction

Hypertension is a preventable contributor with low level of awareness, morbidity, and mortality in the public, which contributes 13.5% proportion of cardiovascular disease-related deaths [1]. Clinic controlling and lowering blood pressure have a significant benefit in reducing the high risk of the hypertensive patients due to the increasing prevalence and its etiologic role in the development of heart attack, stroke, heart failure, and renal failure [2–4]. The integrative medicine and conventional Chinese medicine or herbs on the treatment of hypertension are commonly used and developed as a trend of treatment tool [5]. Chinese medicine contains a variety of compounds, and its advantages from the prescription of its multitarget effects are beneficial in complicated diseases. Hypertension is one of the complicated diseases closely related to the metabonomics of multiple pathways in human body with the biochemical reactions. Recent research of traditional Chinese medicine on hypertension metabonomics mainly depended on ultra performance liquid chromatography combined with time of flight mass spectrum (UPLC-TOF/MS) which possesses high sensitivity, wide dynamic range, and high accuracy [6–11]. There are hypertensive metabonomics studies on Rhizoma Alismatis, Ping Gan prescription, and Tengfu Jiangya tablet, Wistar Kyoto rats (WKYr) and spontaneously hypertensive rats (SHR) were selected as the animal model, and HPLC-TOF-MS was selected as the screening tool to identify the related metabolites. The obtained data was further processed by principal component analysis (PCA) and partial least squares discriminate analysis (PLS-DA) for pattern recognition and selection of significant different content of the metabolites. 12 significantly differential endogenous metabolites and 4 pathways such as purine metabolism, glycerophospholipid metabolism, amino sugar and nucleotide sugar metabolism, and linoleic acid metabolism play important roles in pathogenesis of hypertension and efficacy mechanism of Rhizoma Alismatis. Thirteen biomarkers involved in NO production, inflammatory state, and vascular smooth muscle cells (VSMCs) apoptosis and proliferation, the main metabonomic pathways, were sphingolipid metabolism (sphinganine, lysosphingomyelin, and ceramide), glycerophospholipid metabolism (phosphatidylcholines, phosphatidylethanolamine, and lysophosphatidylcholines), arginine and proline metabolism (l-proline, citrulline), tryptophan metabolism (xanthuiulrenic acid, l-kynurenine, and l-tryptophan), arachidonic acid metabolism (leukotriene D4), and linoleic acid metabolism (gamma-linolenic acid), which could well explain the mechanism of physiological and pathological state of hypertension and the potential therapeutic effects of those prescriptions [12–14].

Qingganqushi** (**QGQS) granule is a widely used Chinese medicine prescription granule (containing Mori Cortex 30 g, Lycii cortex 30 g, Poria 30 g, Stigma Maydis 30 g, Rhizoma Coptidis 6 g, Prunellae spica 30 g, and Pheretima 12 g) to control and lower blood pressure in clinics for hypertension related disease effectively in our hospital by Professor Yuanhui Hu. In particular, QGQS granule has an excellent therapeutic effect on lowing SHR blood pressure by our preliminary research. In this study, SHR was applied to observe characteristics and mechanism of the antihypertensive effect of QGQS granule on hypertension related metabolic syndrome, SHR was administrated with QGQS granule and captopril for 4 weeks, and the rat's blood pressure was compared among model group, control group, captopril group, and QGQS group firstly. Then UPLC-Q-TOF was used to determine and to screen metabolites between SHR and normal rats (WKYr); the differentiated metabolites were analyzed via PCA and PLS-DA methodologies. The vertical signal angiotensin II type 2 receptor ceramide (Ang II-AT2-CE) apoptosis pathway was considered to be responsible for QGQS granule on the treatment of hypertension. When pharmacological indexes of rennin (PRA), angiotensin I (AngI), angiotensin II (AngII), and aldosterone (ALD) were used to verify the pathway analysis by ELISA experiment, a recovery effect of QGQS granule is found on SHR via angiotensin pathway. Related literature showed that the increased level of Ang II may induce the overexpression of profilin-1 in the human umbilical artery smooth muscle cell and in rat aortic tissue of SHR in vitro, the expression level of profilin-1 decreasing could be closely related to the decrease of Ang II [15–17].

We found that Renin-Angiotensin-Aldosterone System (RAAS) was involved in cell proliferation and apoptosis by ceramide (CE), phosphatidyl inositol (PI), which was closely related to the occurrence and development of hypertension via PRA, Ang I, Ang II, and ALD. And it revealed that QGQS granule could inhibit the RAAS effectively to slow down the occurrence and progression of hypertension.

## 2. Materials and Methods

### 2.1. Reagent and Chemicals

Acetonitrile and methanol of chromatographic grade were obtained from Fisher Scientific (Fair Lawn, NJ, USA). Formic acid of HPLC grades was purchased from ROE Scientific Inc. (Newark, DE, USA). ELISA Kit of rat's PRA, Ang I, Ang II, and ALD were all from Shanghai Jianglaibio Co. Ltd. (Shanghai, China). Detection of profiling-1 protein expression kit was containing complete protease inhibitor cocktails from Hoffmann-La Roche Ltd. (Shanghai, China, Lot: 04693116001); PVDF membrane (0.45 *μ*m) was obtained from Millipore (Billerica, MA), SDS-polyacrylamide gel electrophoresis from Sigma-Aldrich Co. Ltd. (Shanghai, China), and nonfat milk from Inner Mongolia Yili Industrial Group Limited by Share Ltd. (Inner Mongolia, China); ECL Western blotting reagents were obtained from Millipore (Billerica, MA, Lot: WBKLS0500). Goat anti rabbit IgG (H + L), bovine serum albumin, horseradish peroxidase (HRP), and goat anti mouse IgG (H + L) were all from Jackson Immuno Research Laboratories, Inc. (West Grove, PA). QGQS granules are composed of Chinese medicine granules Mori cortex (Sangbaipi) 30 g, Lycii cortex (Digupi) 30 g, Poria (Fuling) 30 g, Stigma Maydis (Yumixu) 30 g, Rhizoma Coptidis (Huanglian) 6 g, Prunellae spica (Xiakucao) 30 g, and Pheretima (Dilong) 12 g. The granule was made from Sichuan Neo-Green Pharmaceutical Technology Development Co. Ltd. (Chengdu, China). The prescription was authenticated by Professor Yuanhui Hu (Guang'anmen Hospital China Academy of Chinese Medical Sciences) in clinic for the therapeutic of hypertension. Captopril tablets were purchased from Sino-American Shanghai Squibb Pharmaceuticals Ltd. (Shanghai, China, Lot: AAB7677).

### 2.2. Instrumental

Sample analysis was achieved on a Waters Xevo G2 Q-TOF Mass Spectrometer (TOF-MS), Waters ACQUITY Ultra Performance Liquid Chromatography (UPLC), and an ACQUITY BEH C18 column (50 mm × 2.1 mm, 1.7 *μ*m). All these pieces of equipment were purchased from Waters Corporation (Waters, Milford, MA, USA).

### 2.3. Animal Model and Treatment

The protocol for the animal study was approved by the Animal Experimental Ethical Committee of Guang'anmen Hospital China Academy of Chinese Medical Sciences, and all the efforts were made to ameliorate suffering of animals (number IACUC-GAMH-2015-037). The rats were maintained under the standard laboratory conditions (20 ± 2°C, 45–70% relative humidity, and 12-hour light/dark cycle), with access to standard chow and water ad libitum. All of the rats were purchased from Beijing Vital River Laboratory Animal Technology Co. Ltd. (Beijing, China). The certificate number was SCXK (Beijing) 2015-0001. Fifteen 13-week-aged male (body weight range 200–230 g) and female (body weight range 140–170 g) SHR were used as the model group and experimental group, respectively, five male WKYr (body weight range 300–340 g) and five female WKYr (body weight range 220–240 g) were used as the control group. The 30 SHR were randomly divided into 3 groups (model group, captopril group, and QGQS group) in ten (five male and five female in each group) for each group according to body weight; there was no difference in rats' body weight and rat's blood pressure (RBP) for each group of rats. Model group rats and control group rats were fed with saline water at dose of 1.0 mL per 100 g by body weight. Captopril group rats and QGQS group rats were fed with captopril at dose of 1.5 mg per 100 g and 0.12 g QGQS per 100 g by body weight, respectively. All the rats were fed once a day for 4 weeks.

RBP was measured on tail artery by noninvasive blood pressure measurement consciously with BP-100A rat noninvasive measurement (Chengdu Taimeng Software Co. Ltd., Chengdu, China) at time points of preadministration and after administration of 2, 4, 6, and 8 hours for the first time, and then RBP were determined at 1, 2, 3, and 4 weeks. The RBP was measured three times before administration and after administration for each week. After 4 weeks, the rats were fasting overnight for 12 h, rats were sacrificed under anesthesia, and blood samples were obtained and centrifuged at 3000 ×g for 10 min to obtain serum. Then the serum was transferred into centrifuge tube and stored at −80°C until performing sample procedure for analysis, and the aortic tissues from rats were preserved in liquid nitrogen prior to detecting profiling-1 protein expression.

### 2.4. UPLC-TOF-MS Condition

The plasma sample analysis was carried out on UPLC-TOF-MS system equipped with a waters H-class UPLC system, an ACQUITY BEH C18 column (50 mm × 2.1 mm, 1.7 *μ*m), an electrospray ionization (ESI), and a Q-TOF mass spectrometer. The mobile phases were composed of a mixture of acetonitrile (A) and 0.1% formic acid in water (B) in gradient elution. UPLC-TOF-MS conditions were optimized by parameters of the column temperature, the mobile phases condition, the ESI source temperature, and the drying gas flow rate.

### 2.5. Optimization of Serum Procedure

200 *μ*L of serum was added to a centrifuge tube; methanol or acetonitrile volumes were investigated at ratio of 1 : 1, 1 : 2, 1 : 3, 1 : 4, and 1 : 5 (v/v) to precipitate the protein. The extraction solvent was investigated with addition to formic acid and the concentration. Then the sample mixture was shaken vigorously for 2 min prior to keeping at 4°C for 10 minutes. The sample mixture was centrifuged at 15000 ×g for 20 min to get supernatant. After centrifugation, 800 *μ*L of the supernatant was transferred into a centrifuge tube by repeated centrifugation once. The supernatant was placed into sample vial under 4°C before analysis.

### 2.6. ELISA Assay for Pathway Confirmatory

The serum samples ELISA measurement was conducted according to commercial ELISA kits instructions to determine the pharmacological indexes of PRA, Ang I, Ang II, and ALD. The absorbance of each sample was measured on a Lab systems Multiskan MS 352 microreader (Thermo Fisher Scientific Inc., USA). The concentrations of PRA, Ang I, Ang II, and ALD were calculated based on each calibration curve, respectively.

### 2.7. WB Determination of Profilin-1 Protein Expression

The rat thoracic aorta peeling in each group was used for profilin-1 protein expression by Western blotting (WB) experiment. The RIPA protein extraction reagent was precooled at 4°C and then added to protease inhibitor cocktail (Hoffmann-La Roche Ltd., China). 10 mg of the homogenized rat thoracic aorta tissue was added to 100 *μ*L of the prepared RIPA protein extraction reagent prior to extracting eventually; then the sample was incubated under 0°C for 20 min and subsequently centrifuged at 13,000 ×g for 20 min at 4°C. The catalyzed samples were resolved by SDS-polyacrylamide gel electrophoresis and then transferred onto a PVDF membrane. Blots were blocked for 1 h at 25°C by 5% Tris-buffered saline-Tween (TBST) containing 5% bovine serum albumin (TBST-BSA). The membranes were washed with goat anti rabbit IgG (H + L), HRP, and goat anti mouse IgG (H + L), HRP (1 : 10000), which were incubated overnight at 4°C in 5% TBST-BSA solution. The PVDF membrane was washed with TBST for 10 min in 3 times. ECL Western blotting reagents were added dropwise to the reaction surface membrane protein for 3 min; then the blot was exposed to X-ray film to obtain a reaction strip. The gray strips were quantified using a gel image system ver. 4.00 (Tanon Science & Technology Co. Ltd., Shanghai, China).

### 2.8. Statistical Analysis

All the data was analyzed using Microsoft Excel 2007 and SPSS Statistics 17.0 (SPSS, USA). RBP data was expressed by mean and standard deviation (*x* ± SD). The level of significance among each group was analyzed by one-way ANOVA and *t*-test, where *p* < 0.05 was considered to be significant difference, while *p* < 0.01 was considered to be very significant difference. UPLC-Q-TOF-MS data collection system was equipped with Waters Progenesis Qi software (Milford, MA, USA) to transform the raw data into a single matrix containing aligned peaks. The matrix was introduced to EZinfo 2.0 software for PCA and PLS-DA analysis. The obtained *m*/*z* results of PCA or OPLS-DA were displayed as score plots that represent the scatter of each sample. According to the loading plot, tightly clustered plots indicated similar metabolic phenotypes. The purpose of PLS-DA was used to develop models that differentiated potential pathway between drug treatment group and model group. The variable importance in projection (VIP) value produced by PLS-DA was applied to find potential biomarkers; the variable with VIP values > 1 was considered to be significant difference.

## 3. Results

### 3.1. RBP Testing

**Figure 1:**
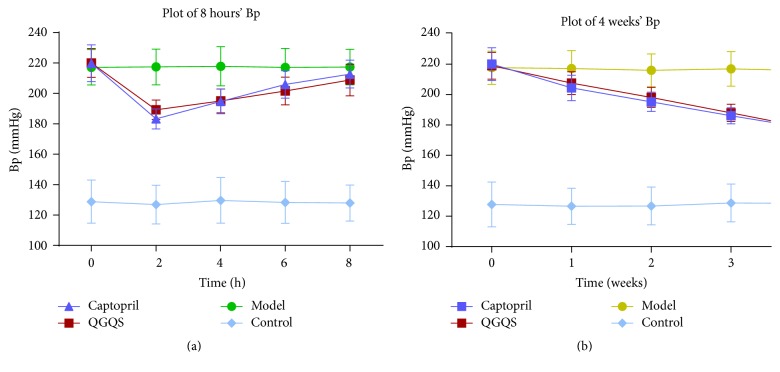
The RBP testing results plot at time points of 0, 2, 4, 6, and 8 h and 0 to 4 weeks in each group of rats.

The RBP in each group is displayed in Figure 1. After SHR was administrated with QGQS granule or captopril, the RBP was gradually decreased from 0 h to 2 h, while RBP was increased gradually after 2, 4, 6, and 8 hours for the first time. There was a very significant difference of RBP in QGQS group and captopril group (*p* < 0.01) compared with model group at time points of 2, 4, 6, and 8 h. There was no significant difference of RBP between QGQS group and captopril group. After SHR was administrated with QGQS granule or captopril from 0 to 4 weeks, the RBP of two groups were gradually decreased. There was no significant difference of RBP between QGQS group and captopril group within those 4 weeks. The RBP of QGQS group and captopril group were very significantly decreased compared with model group (*p* < 0.01).

### 3.2. UPLC-Q-TOF-MS Method Condition

For the UPLC system, the column temperature was optimized at 40°C. The autosampler was maintained at 4°C, and the injection volume was 3 *μ*L. The mobile phases were a mixture of acetonitrile (A) and 0.1% formic acid in water (B) at a flow rate of 0.3 mL·min^−1^. The gradient elution program was 0–2 min, A (5–10%) versus (versus) B (95–90%), 2–15 min, A (50–100%) versus B (50–0%), 15–17 min, and A (100–5%) versus B (0–95%). For the Q-TOF mass spectrometer, ESI source was operated in both positive ion mode and negative ion mode, the ion source temperature was at 100°C, the parameter for cone voltage was 40 kV, and capillary voltage was 2.5 kV. The desolvation gas was at a flow rate of 800 L·h^−1^ under temperature of 400°C; the cone gas was at a flow rate of 50 L·h^−1^. MS data collections were carried out at dynamic range of 50 to 1000* m/z* with scanning interval of 0.02 seconds. Leucine-Enkephalin for Lock-spray was used to ensure the quality of repeatability and accuracy.

### 3.3. Sample Procedure

After optimizing the serum extraction method for UPLC-MS analysis, sample procedure was as follows: an aliquot of 800 *μ*L methanol was added to 200 *μ*L serums to precipitate the protein in a centrifuge tube. The mixture was shaken vigorously for 2 min, then kept at 4°C for 10 minutes, and then centrifuged at 15000 ×g for 20 min to obtain the supernatant. After that, 800 *μ*L of supernatant was transferred into a new centrifuge tube prior to centrifuging at 15000 ×g for 20 min to obtain the supernatant. The final obtained supernatant was transferred into the sample vial under 4°C for analysis.

### 3.4. UPLC-TOF Data Acquisition

**Figure 2:**
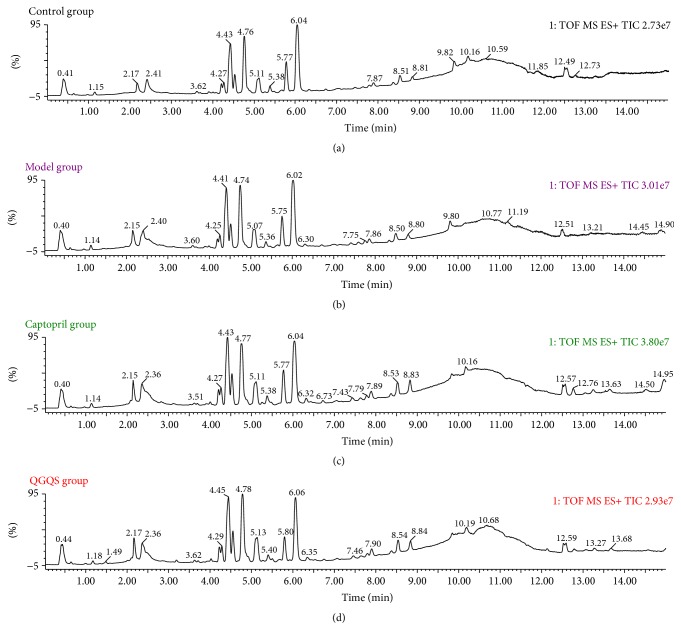
The typical ESI+ TIC of control group, model group, captopril group, and QGQS group.

The serum samples were analyzed in control group, model group, captopril group, and QGQS group. The total ion chromatography (TIC) was obtained. The typical TIC for each group was presented at Figure 2. The potential small-molecule metabolites (*m*/*z*) were extracted in both positive and negative ion model.

### 3.5. PLS-DA Analysis

**Figure 3:**
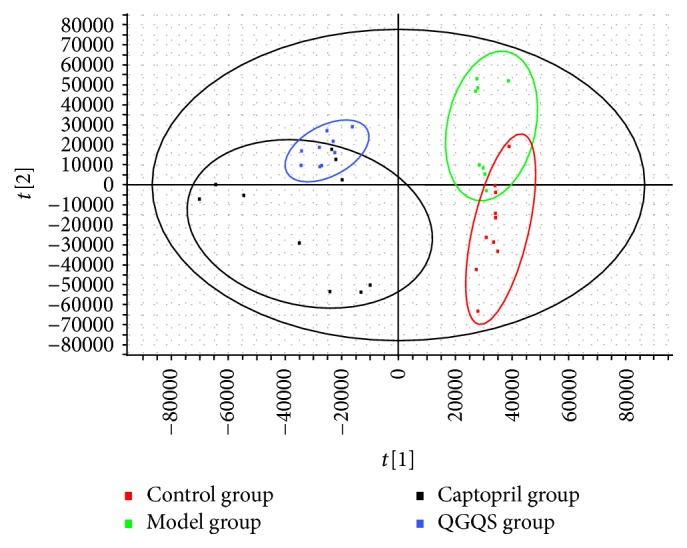
The plotted points from ESI+ of control group, model group, captopril group, and QGQS group.

**Figure 4:**
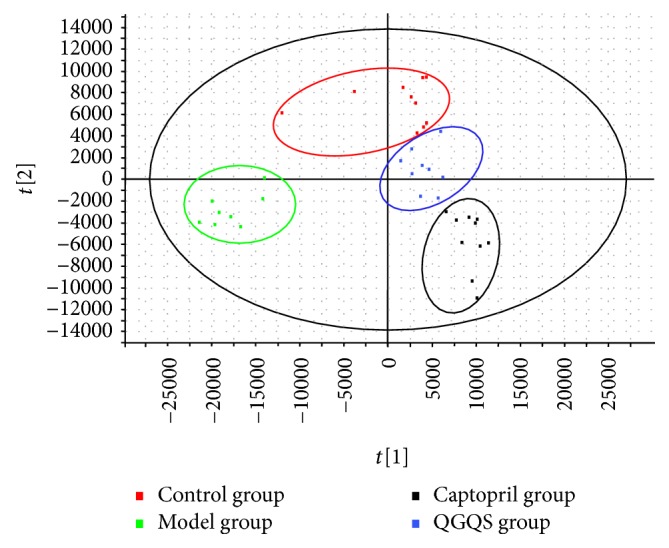
The plotted points from ESI− of control group, model group, captopril group, and QGQS group.

The PLS-DA score plot of the positive mode (Figure 3) and the negative mode (Figure 4) clearly illuminated the distribution of the potential biomarkers among the control group, model group, captopril group, and QGQS group. After QGQS granule or captopril intervention for 8 weeks, the plotted points of ESI+ were quite different in control group from model group; QGQS group was close to control group and away from the model group. The plotted points of ESI− were very close to the control group of the normal rats, which suggested that the changed SHR metabolic pattern was close to health condition. And captopril group seemed to be away from the control group compared with QGQS group in ESI− plotted points, which indicated that QGQS granule may perform better than captopril in metabolic amelioration for hypertension.

### 3.6. Potential Biomarker and Metabolism Pathway Analysis

**Figure 5:**
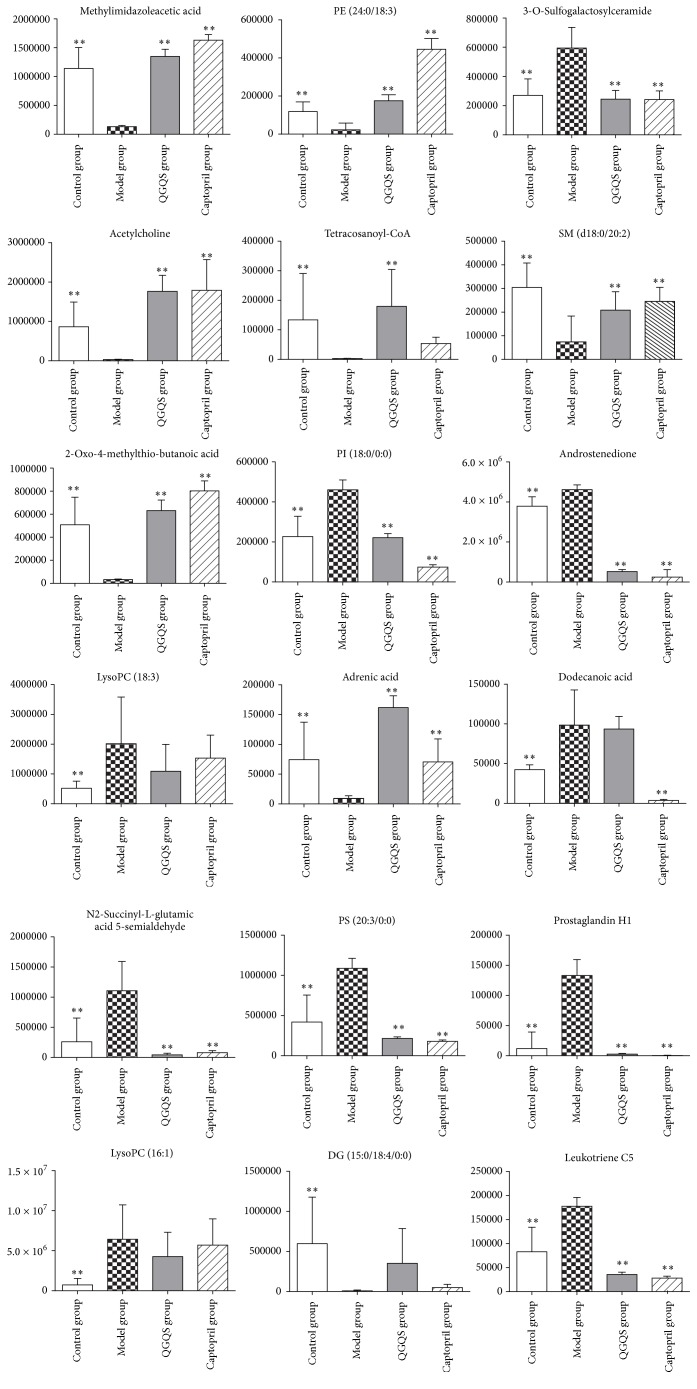
The comparison of the potential metabolites from ESI− and ESI+ of control group, model group, captopril group, and QGQS group. *∗∗* is the representation that there is a very significant difference between model group and control group and captopril group or QGQS group (*p* < 0.01).

After processing the data of PCA and PLS-DA analysis, VIP scores for each variable were obtained (Table 1); higher VIP scores meant increasing importance in the predictive model. The metabolites and metabolic pathways were further identified based on accurate molecular weight and MS spectra and confirmed by METLIN (http://metlin.scripps.edu/) and HMDB (http://www.hmdb.ca/) database. Ultimately, 20 potential biomarkers and their trends were identified, such as LysoPC (18:3), LysoPC (16:1), PE (24:0/18:3), PI (18:0/0:0), PS (20:3/0:0), PC (15:0/0:0), methylimidazoleacetic acid, 2-Oxo-4-methylthiobutanoic acid, N2-succinyl-L-glutamic acid 5-semialdehyde, tetracosanoyl-CoA, adrenic acid, dodecanoic acid, leukotriene C5, 3-O-sulfogalactosylceramide (d18:1/16:0), SM (d18:0/20:2), acetylcholine, diacylglycerol, androstenedione, and prostaglandin H1. The MS intensity of the metabolites comparisons among each group was shown in Figure 5.

### 3.7. ELISA Verification Pathway of Angiotensin Inhibition Effect

The indexes of PRA, Ang I, Ang II, and ALD were obtained in each group by ELISA assay verification. In model group, the levels of those indexes were significantly more than control group. After treatment with QGQS granule or captopril for 4 weeks, those indexes were significantly reduced compared with the model group (*p* < 0.01) while significantly more than control group. There was no significant difference between groups of captopril and QGQS granule (Table 2).

### 3.8. WB Determination of the Expression on Profilin-1 Protein

**Figure 6:**
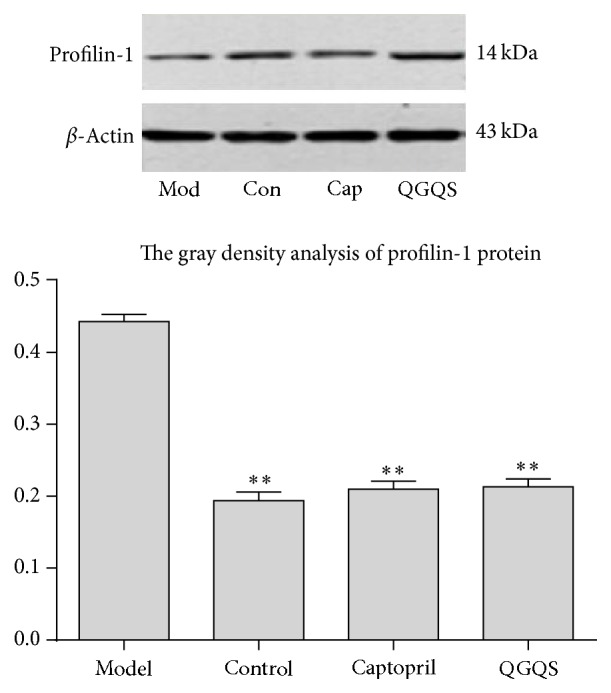
The profilin-1 protein expression of control group, model group, captopril group, and QGQS group. *∗∗* is the representation of *p* < 0.01 of model group compared with control group and QGQS group or captopril group (*p* < 0.01).

Quantification of profilin-1 protein expression in aortic tissue was shown in Figure 6 for each group. In model group, the levels of profilin-1 protein expression were significantly much more than control group, QGQS group, or captopril group (*p* < 0.01). After being treated with QGQS granule or captopril for 4 weeks, the profilin-1 protein expression was significantly reduced in QGQS group or captopril group, while there was no difference for the profilin-1 protein expression from QGQS group compared with captopril group. These results suggested that QGQS granule or captopril may inhibit the overexpression of profilin-1.

## 4. Discussion

### 4.1. Efficacy of QGQS Granule on SHR Blood Pressure

This study shows that the therapeutic effect QGQS of granule on hypertension is similar to captopril from 0 to 4 weeks; the variety of RBP of QGQS group from postadministration at time points of 2, 4, 6, and 8 hours is less than captopril group, while the final RBP of QGQS group is no more than captopril group. The results display the advantage of QGQS granule in therapeutic of SHR much better than captopril.

### 4.2. Effect on Lipid and Arachidonic Acid Metabonomics

Lysophosphatidylcholine (LysoPC) is one of the important components for oxidized low-density lipoprotein (ox-LDL), mild modified low-density lipoprotein (mm-LDL), which is synthesized from phospholipids under catalysis of lecithin cholesterol acyltransferase [18]. LysoPC have effect on increasing the permeability of vascular endothelial cells proliferation, migration, and apoptosis of VSMCs and bidirectional transportation of cholesterol from foam cells. LysoPC is one of the major lipid components in damage of vascular endothelium via influencing the synthesis and release of nitric oxide (NO) [19–24]. Literature indicates that sphingomyelin (SM), phosphatidylcholine (PC), and phosphatidylserine (PS) increase while phosphatidyl ethanolamine (PE) decrease occurred among different stage patients with hypertension; those phospholipids in plasma are mainly derived from blood platelets and erythrocyte membrane phospholipids. PS is a cofactor of coagulation accelerator by promoting hemagglutination and intercellular contact to increase blood viscosity and blood pressure. The decrease of PC and increase of PE suggest the oxidation resistance increase [25]. This is one of the pathways for QGQS granule action.

3-o-Sulfogalactosylceramide is one of metabolic intermediates on CE signaling pathway, an important intracellular of secondary messenger and one of the components of myocardial cell membrane [26], which has important functions in regulating cardiomyocytes proliferation, differentiation, and apoptosis [27–30]. CE could activate protein kinase C (PKC) and c-Jin to induce vascular endothelial cell apoptosis. CE keeps the balance of vasoconstrictors and vasodilators, which are constricted vasoconstriction, vascular stenosis, and high blood pressure. CE activates stress-activated protein kinases/c-JUN N-terminal protein kinase (SAPK/JNK); SAPK/JNK leads to activator protein-1 (AP-1) transcription factor phosphorylation, which is a regulator of activating transcription factor to stimulate self-expression, enhance its activity, and mediate the related cell apoptosis to affect blood pressure [31]. Ang II activates phospholipase C, the activated phospholipase C can promote PI metabolism to produce phosphatidic acid (PA), and in the synergistic effect of Ca^2+^, the arachidonic acid is separated immediately from the phospholipid membrane before metabolism [32].

Leukotriene and prostaglandin are metabolites originating from arachidonic acid, which involves inflammation and immune-reaction [33]. Experiment shows that the lack of prostaglandin I2 (PGI2) receptor in mice will gradually develop hypertension and left ventricular hypertrophy [34]. Arachidonic acid can affect blood pressure via regulating the content and proportion of thromboxane A2 (TXA2) and PGI2 [35]. TXA2 is involved in vasoconstriction, while PGI2 is involved in vasodilatation; both of them maintain a homeostatic balance [36]. Arachidonic acid modulates blood pressure primarily by affecting the content and proportion of TXA2 and PGI2 [37]. Leukotrienes are involved in the pathogenesis of hypertension by promoting monocyte migration and adhesion to endothelial cells, transforming foam cells and macrophages, activating lymphocytes, promoting vascular smooth muscle cell proliferation, and stimulating arterial contraction occurrence and development [38].

**Figure 7:**
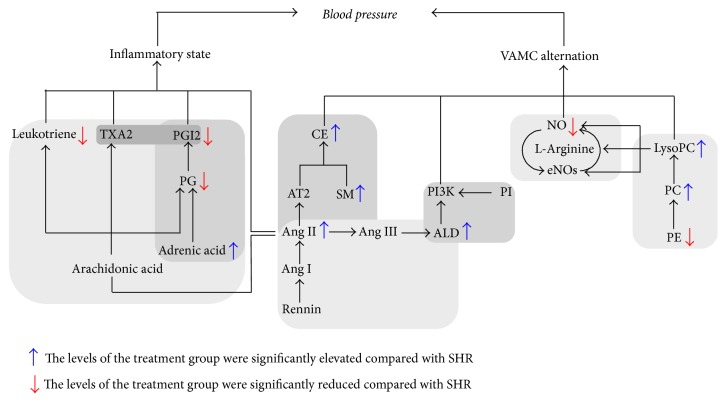
The metabolites and the metabolic pathways analysis of QGQS granule.

Our research indicates that QGQS granule and captopril could inhibit occurrence and development of hypertension by downregulating expression of LysoPC, PS, CE, and leukotriene, while the difference between QGQS granule and captopril is the content of adrenic acid (known as docosatetraenoic acid), which is significantly more in QGQS group than in captopril group. Adrenic acid could inhibit the formation and activity of PG, relax coronary artery, and regulate adrenal artery vascular tone to help vasodilation by intervention from vascular endothelium and the metabolites of ZG cells CYP450 enzyme [39, 40]. From the metabonomics analysis, the serum potential biomarkers of QGQS granule on SHR should be mainly focused on the metabolism of glycerophospholipid, sphingolipid, and arachidonic acid. The metabolites and the metabolic pathways analyses are shown in Figure 7.

### 4.3. Inhibition of Angiotensin Pathway Verification

Acetylcholine plays a major role in the cardiovascular system of the dilation of blood vessels, heart rate, negative conduction, and decrease myocardial contractility [41, 42], which could activate presynaptic M1 receptor in sympathetic nerve endings, thereby inhibiting the nerve ending release of noradrenaline (NA) to make antihypertensive effect on blood vessel [43]. Acetylcholine, as the common neurotransmitter substance of the central and peripheral nerve system, is a special way to inhibit angiotensin. Ang II could induce human umbilical vein endothelial cells apoptosis by activation of AT2, which has effect on phospholipids metabolism and CE synthesis. That is the formation of the vertical signal Ang II-AT2-CE apoptosis pathway.

### 4.4. Inhibitory Effect on Profilin-1

The above results indicate that under the condition of hypertension, the level of Ang II may induce the overexpression of profilin-1, which is involved in cell proliferation and apoptosis of the aortic tissue related cell. QGQS granule takes effect on the overexpression of profilin-1 protein, which have equal effect compared with captopril.

Rennin is secreted from juxtaglomerular cell, which could activate liver to produce Ang I, and then transferred into Ang II, and Ang III transferred from Ang II. Ang III can stimulate adrenal cortex to synthesize and release ALD. Ang II is the most important ingredient, which acts on the heart and blood vessels of the Ang II receptor subtype 1 (AT1); a direct contraction of small arteries promotes the increased sympathetic nerve endings release of catecholamines to promote high blood pressure [44]. Besides, Ang II is one of the inflammatory mediators involved in all the steps of vascular inflammation, such as proinflammatory cytokines inducing cells to produce reactive oxygen, cytokines, and adhesion molecules involved in inflammation and activating redox-sensitive inflammation gene involved in inflammation [45, 46]. Reactive oxygen species (ROS) is induction by Ang II (NADPH oxidase). And the activated xanthine oxidase (XOD) is also produced by Ang II to induce ROS, which caused oxidative stress to aggravate endothelial cell apoptosis [47–49].

Protein-phosphatidylinositol-3-kinase (PI3K) is originated from phosphorylation of PI and involved in regulation of various cellular biological processes, such as cell proliferation and apoptosis, cell migration, protein synthesis, and metabolism [50]. ALD could cause action proteins changes by activating PI3K and mitogen-activated protein kinas (MAPK). This nondependent effect on Ang I is activated and exerted effect on the protein synthesis, cell proliferation, and apoptosis inhibition [49].

In this study, QGQS granule shows an excellent therapeutic effect on hypertension of SHR; it is more stable and sustained compared with captopril for the treatment of hypertension in control of blood pressure. Meanwhile, UPLC-Q-TOF technology is applied to screen metabonomics and metabolites between WKYr control rats, SHR, QGQS group rats, and captopril group rats; metabolism of glycerophospholipid, sphingolipid, and arachidonic acid for metabonomics is differentiated in each group. A recovery effect of QGQS granule on SHR was verified via the angiotensin pathway analysis by ELISA experiment. We confirmed that RAAS was involved in cell proliferation and apoptosis by CE, PI, which was closely related to the occurrence and development of hypertension via PRA, Ang I, Ang II, and ALD. And the study indicated that QGQS granule could inhibit the RAAS effectively to slow down the progression of hypertension.

## 5. Conclusion

In conclusion, QGQS granule has an excellent therapeutic effect on hypertension. It is more stable and sustained compared with captopril on lowing blood pressure. The metabonomics technology could be applied to identify and distinguish the metabolites in SHR and healthy WKYr (identified 19 types of the metabolites), and metabonomic pathway is mainly related to the metabolism of glycerophospholipid, sphingolipid, and arachidonic acid. After ELISA experiment verification, RAAS should be responsible for therapeutic of hypertension of QGQS granule.

## Figures and Tables

**Table 1:** UPLC-Q-TOF indicated metabolites and potential pathway analysis.

Number	*t*_*R*_ (min)	*m*/*z*	VIP	*p* value	Formula	Indicated metabolites	Pathway
(1)	0.37	175.0282	2.32	0.00001	C_6_H_8_N_2_O_2_	Methylimidazoleacetic acid	Histidine metabolism
(2)	0.64	167.0938	2.9	0.0001	C_7_H_16_NO_2_^+^	Acetylcholine	Acetylcholine synthesis
(3)	1.31	212.0549	2.02	0.0002	C_9_H_13_NO_6_	N2-Succinyl-L-glutamic acid 5-semialdehyde	Arginine and proline metabolism
(4)	2.22	498.3017	3.27	0.014	C_26_H_48_NO_7_P	LysoPC (18:3)	Glycerophospholipid metabolism
(5)	2.26	514.2937	5.72	0.001	C_24_H_48_NO_7_P	LysoPC (16:1)	Glycerophospholipid metabolism
(6)	2.59	860.6016	1.44	0.0001	C_47_H_88_NO_8_P	PE (24:0/18:3)	Glycerophospholipid metabolism
(7)	4.16	619.4636	1.64	0.005	C_36_H_62_O_5_	DG (15:0/18:4/0:0)	Diacylglycerol metabolize
(8)	4.19	1138.4354	1.04	0.001	C_45_H_82_N_7_O_17_P_3_S	Tetracosanoyl-CoA	Biosynthesis of unsaturated fatty acids
(9)	4.24	500.2908	2.13	0.0001	C_23_H_48_NO_6_P	PC (P-15:0/0:0)	Glycerophospholipid metabolism
(10)	4.48	660.2416	1.82	0.0002	C_30_H_45_N_3_O_9_S	Leukotriene C5	Arachidonic acid metabolism
(11)	4.7	353.2345	2.26	0.0001	C_20_H_34_O_5_	Prostaglandin H1	Prostaglandin synthesis and regulation
(12)	4.82	592.2889	1.89	0.0001	C_26_H_46_NO_9_P	PS (20:3/0:0)	Glycerophospholipid metabolism
(13)	4.87	353.2461	1.29	0.0002	C_22_H_36_O_2_	Adrenic acid	Biosynthesis of unsaturated fatty acids
(14)	5.13	583.3287	1.28	0.0001	C_27_H_53_O_11_P	PI (18:0/0:0)	Glycerophospholipid metabolism
(15)	7.3	799.5952	1.24	0.004	C_43_H_83_N_2_O_6_P	SM (d18:0/20:2)	Sphingolipid metabolism
(16)	8.83	129.0020	1.6	0.0001	C_5_H_8_O_3_S	2-Oxo-4-methylthio-butanoic acid	Methionine metabolism
(17)	11.27	779.5189	1.25	0.0001	C_40_H_77_NO_11_S	3-O-Sulfogalactosylceramide (d18:1/16:0)	Sphingolipid metabolism
(18)	11.88	479.2471	2.66	0.0001	C_24_H_40_O_6_S	Lithocholic acid sulfate	Biological oxidations
(19)	13.44	237.1266	1.14	0.06	C_19_H_26_O_2_	Dodecanoic acid	Fatty acid biosynthesis
(20)	14.9	595.3736	2.44	0.0003	C_19_H_26_O_2_	Androstenedione	Steroid biosynthesis

**Table 2:** ELISA results of ALD, PRA, Ang I, and Ang II in each group.

Group	PRA (ng/L)	Ang I (ng/L)	Ang II (ng/L)	ALD (ng/L)
Captopril	143.09 ± 12.78^*∗∗*^	809.43 ± 38.06^*∗∗*^	325.59 ± 29.10^*∗∗*^	310.89 ± 37.71^*∗∗*^
QGQS	145.70 ± 15.07^*∗∗*^	820.65 ± 38.42^*∗∗*^	332.80 ± 29.01^*∗∗*^	314.56 ± 38.30^*∗∗*^
Model	197.78 ± 11.20	945.42 ± 25.00	394.05 ± 28.24	387.01 ± 24.17
Control	111.05 ± 7.21	628.37 ± 39.47	231.05 ± 28.78	260.26 ± 33.34

*∗∗* is the representation of *p* < 0.01 in drug group compared with model group.
